# Supplementary material for: A Novel Predictive Multi-Marker Test for the Pre-Surgical Identification of Ovarian Cancer
Source: Cancers (Basel). 2023 Nov 2;15(21):5267. doi: 10.3390/cancers15215267 (PMC10650329; doi:10.3390/cancers15215267)
Supplement: Supplementary file 1 [file cancers-15-05267-s001.zip › cancers-2691689-supplementary.pdf]

Sample #	AGE at collection	menopausal status	Genetic predisposition (BRCA1/2/WT/INC; LYNCH; BRIP1; RAD51C; VUS)	Benign / Malignant	Pathology type	Tumour grade	Tumour Stage
**8**	21.2	pre	unknown	**Benign**	fibroma	n/a	n/a
**29**	27.8	pre	unknown	**Benign**	mature teratoma	n/a	n/a
**30**	46.2	pre	unknown	**Benign**	cystadenomata	n/a	n/a
**42**	27.6	pre	unknown	**Benign**	hydrosalpinx	n/a	n/a
**52**	25.3	pre	unknown	**Benign**	fibroma	n/a	n/a
**135**	43.0	pre	unknown	**Benign**	ectopic pregnancy and haemorrhage	n/a	n/a
**226**	37.3	pre	unknown	**Benign**	endometrial hyperplasia with no evidence of malignancy	n/a	n/a
**287**	76.0	pre	unknown	**Benign**	Xanthogranulomatous	n/a	n/a
**320**	47.1	pre	unknown	**Benign**	cystadenoma	n/a	n/a
**343**	51.1	pre	unknown	**Benign**	fibroma	n/a	n/a
**344**	46.3	pre	unknown	**Benign**	mature teratoma	n/a	n/a
**356**	31.4	pre	unknown	**Benign**	Torted infarcted ovarian cystadenoma	n/a	n/a
**385**	42.8	pre	unknown	**Benign**	cyst-dermoid	n/a	n/a
**847**	49.2	pre	unknown	**Benign**	Lipoid cell tumour	n/a	n/a
**856**	44.9	pre	unknown	**Benign**	cystadenoma	n/a	n/a
**884**	46.1	pre	unknown	**Benign**	cystadenoma	n/a	n/a
**1023**	46.4	pre	unknown	**Benign**	cyst	n/a	n/a
**1106**	39.6	pre	unknown	**Benign**	ovarian endometriosis	n/a	n/a
**1132**	48.4	pre	BRCA1+	**Benign**	cyst, cervical metaplasia	n/a	n/a
**1139**	42.5	pre	BRCA2+	**Benign**	cervical metaplasia	n/a	n/a
**1192**	46.8	pre	BRCA2+	**Benign**	H corpus	n/a	n/a
**1203**	44.0	pre	BRCA1+	**Benign**	cyst	n/a	n/a
**1229**	38.7	pre	LYNCH	**Benign**	cervical metaplasia	n/a	n/a
**1247**	55.8	pre	BRCA2+	**Benign**	cyst	n/a	n/a
**1330**	46.5	pre	BRCA2+	**Benign**	uterine leiomyomata	n/a	n/a
**1380**	38.1	pre	BRCA1+	**Benign**	uterine adenomyosis	n/a	n/a
**1383**	48.8	pre	unknown	**Benign**	uterine adenomyosis	n/a	n/a
**1403**	40.4	pre	BRCA1+	**Benign**	salpingitis and cyst	n/a	n/a
**1438**	39.8	pre	BRCA2+	**Benign**	H corpus, cyst	n/a	n/a
**1444**	43.3	pre	BRCA1+	**Benign**	uterine leiomyomata	n/a	n/a
**1457**	43.5	pre	LYNCH	**Benign**	uterine leiomyoma	n/a	n/a
**1470**	52.5	pre	unknown	**Benign**	adenofibroma	n/a	n/a
**1506**	46.8	pre	BRCA2+	**Benign**	salpingitis	n/a	n/a
**1517**	47.4	pre	LYNCH	**Benign**	cyst	n/a	n/a
**1538**	41.3	pre	BRCA1+	**Benign**	benign changes	n/a	n/a
**1542**	40.2	pre	BRCA2+	**Benign**	cyst	n/a	n/a
**1562**	40.5	pre	BRCA1+	**Benign**	cyst	n/a	n/a
**1572**	41.1	pre	BRCA1+	**Benign**	cyst	n/a	n/a
**1587**	42.3	pre	LYNCH	**Benign**	uterine leiomyoma	n/a	n/a
**1595**	37.6	pre	BRCA2+	**Benign**	benign changes	n/a	n/a
**1605**	46.7	pre	BRCA WILDTYPE	**Benign**	mullerian cystadenoma	n/a	n/a
**1615**	49.9	pre	BRCA2+	**Benign**	cyst	n/a	n/a
**1622**	50.2	pre	BRCA1+	**Benign**	uterine adenomyosis	n/a	n/a
**1671**	48.4	pre	BRCA2+	**Benign**	cyst	n/a	n/a
**1707**	46.3	pre	BRCA2+	**Benign**	fibrous adhesions	n/a	n/a
**1723**	50.9	pre	unknown	**Benign**	cyst	n/a	n/a
**1750**	34.1	pre	BRCA2+	**Benign**	cyst	n/a	n/a
**1754**	50.8	pre	unknown	**Benign**	ovarian endometriosis, cyst	n/a	n/a
**1771**	47.1	pre	BRCA1+	**Benign**	uterine leiomyoma	n/a	n/a
**1788**	42.1	pre	RADBRIP1+1C+	**Benign**	ovarian endometriosis	n/a	n/a
**1791**	48.6	pre	BRCA2+	**Benign**	uterine leiomyoma	n/a	n/a
**1796**	48.3	pre	PALB2+	**Benign**	seromucinous cystadenoma	n/a	n/a
**1802**	40.5	pre	BRCA1+	**Benign**	cyst, uterine leiomyoma	n/a	n/a
**1803**	42.8	pre	BRCA2+	**Benign**	calcifications	n/a	n/a
**1806**	44.2	pre	BRCA1+	**Benign**	benign changes	n/a	n/a
**1839**	46.3	pre	Lynch syndrome	**Benign**	cyst H corpus	n/a	n/a
**1854**	41.5	pre	BRCA1+	**Benign**	benign changes	n/a	n/a
**1884**	36.7	pre	RADBRIP1+1C+	**Benign**	cyst	n/a	n/a
**1900**	41.5	pre	BRCA2+	**Benign**	uterine adenomyosis	n/a	n/a
**1914**	50.0	pre	BRCA2+	**Benign**	H corpus	n/a	n/a
**1919**	40.2	pre	RADBRIP1+1C+	**Benign**	benign changes	n/a	n/a
**1950**	38.9	pre	Lynch syndrome	**Benign**	cyst, uterine adenomyosis	n/a	n/a
**1956**	38.3	pre	BRCA1+	**Benign**	uterine leiomyoma, cervical metaplasia	n/a	n/a
**1973**	40.4	pre	BRCA2+	**Benign**	cyst	n/a	n/a
**1975**	42.3	pre	BRCA1+	**Benign**	cyst	n/a	n/a
**EP0020**	45.3	pre	LYNCH	**Benign**	cyst	n/a	n/a
**EP0052**	31.4	pre	BRCA1+	**Benign**	cyst	n/a	n/a
**EP0116**	46.1	pre	BRCA WILDTYPE	**Benign**	cyst	n/a	n/a
**EP0196**	47.2	pre	unknown	**Benign**	uterine adenomyosis, cervical metaplasia	n/a	n/a
**EP0209**	43.1	pre	BRCA1+	**Benign**	H corpus	n/a	n/a
**EP0244**	50.1	pre	LYNCH	**Benign**	uterine leiomyoma	n/a	n/a
**EP0275**	53.4	pre	unknown	**Benign**	bladder papilloma benign	n/a	n/a
**EP0276**	36.3	pre	BRCA wildtype	**Benign**	ovarian endometriosis	n/a	n/a
**EP0308**	41.8	pre	BRCA2+	**Benign**	H corpus and cyst	n/a	n/a
**EP0312**	49.6	pre	BRCA1+	**Benign**	cyst	n/a	n/a
**EP0348**	50.2	pre	BRCA WILDTYPE	**Benign**	cyst and salpingosis	n/a	n/a
**EP0349**	33.4	pre	BRCA2+	**Benign**	ovarian endometriosis and cyst	n/a	n/a
**EP0353**	43.7	pre	BRCA2+	**Benign**	H corpus	n/a	n/a
**EP0371**	47.1	pre	unknown	**Benign**	cyst	n/a	n/a
**EP0383**	38.2	pre	BRCA2+	**Benign**	serous cystadenoma	n/a	n/a
**EP0399**	35.8	pre	BRCA1+	**Benign**	cyst	n/a	n/a

Sample #	AGE at collection	menopausal status	Genetic predisposition (BRCA1/2/WT/INC; LYNCH; BRIP1; RAD51C; VUS)	Benign / Malignant	Pathology type	Tumour grade	Tumour Stage
**EP0411**	44.3	pre	BRCA1+	**Benign**	cyst	n/a	n/a
**EP0467**	39.7	pre	BRCA1+	**Benign**	cyst	n/a	n/a
**26**	73.7	post	unknown	**Benign**	Cystadenoms	n/a	n/a
**31**	55.6	post	unknown	**Benign**	cystadenoma	n/a	n/a
**41**	65.0	post	Unknown	**Benign**	adenofibroma	n/a	n/a
**47**	55.4	post	unknown	**Benign**	cystadenoma	n/a	n/a
**98**	50.6	post	unknown	**Benign**	simple cyst	n/a	n/a
**140**	73.8	post	unknown	**Benign**	mucinous fibroma and benign Brenner tumour	n/a	n/a
**352**	74.3	post	unknown	**Benign**	Haemorrhagic ovarian torsion	n/a	n/a
**512**	57.7	post	unknown	**Benign**	simple cyst	n/a	n/a
**560**	52.4	post	Unknown	**Benign**	fibroma	n/a	n/a
**615**	82.5	post	unknown	**Benign**	cystadenoma	n/a	n/a
**620**	63.6	post	unknown	**Benign**	fibrothecoma	n/a	n/a
**646**	82.5	post	Unknown	**Benign**	fibroma	n/a	n/a
**723**	87.4	post	unknown	**Benign**	cystadenofibroma	n/a	n/a
**753**	62.2	post	unknown	**Benign**	cystadenoma	n/a	n/a
**771**	87.1	post	unknown	**Benign**	fibroma	n/a	n/a
**775**	54.1	post	unknown	**Benign**	Papillary adenofibroma of the right ovary	n/a	n/a
**788**	57.1	post	unknown	**Benign**	fibroma	n/a	n/a
**814**	60.2	post	unknown	**Benign**	fibroma	n/a	n/a
**843**	80.7	post	unknown	**Benign**	cystadenofibroma	n/a	n/a
**879**	56.1	post	unknown	**Benign**	cystadenoma	n/a	n/a
**883**	78.4	post	Unknown	**Benign**	fibroma	n/a	n/a
**922**	67.4	post	unknown	**Benign**	fibroma	n/a	n/a
**996**	61.9	post	BRCA2+	**Benign**	salpingitis	n/a	n/a
**1082**	57.2	post	BRCA1+	**Benign**	serous adenofibroma& brenner tumour	n/a	n/a
**1092**	54.2	post	unknown	**Benign**	cyst	n/a	n/a
**1103**	49.7	post	unknown	**Benign**	H corpus & cyst	n/a	n/a
**1131**	68.2	post	BRCA1+	**Benign**	cyst	n/a	n/a
**1209**	61.3	post	unknown	**Benign**	cyst	n/a	n/a
**1288**	60.4	post	BRCA2+	**Benign**	benign changes	n/a	n/a
**1289**	66.0	post	BRCA1+	**Benign**	benign changes	n/a	n/a
**1307**	55.7	post	unknown	**Benign**	benign changes	n/a	n/a
**1312**	48.5	post	BRCA1+	**Benign**	cyst	n/a	n/a
**1338**	47.9	post	BRCA1+	**Benign**	salpingitis	n/a	n/a
**1376**	67.7	post	BRCA1+	**Benign**	stromal and leydig hyperplasia	n/a	n/a
**1377**	75.4	post	LYNCH	**Benign**	uterine adenomyosis	n/a	n/a
**1387**	54.7	post	BRCA2+	**Benign**	Benign changes	n/a	n/a
**1414**	63.3	post	unknown	**Benign**	cyst	n/a	n/a
**1422**	69.6	post	BRCA1+	**Benign**	cyst	n/a	n/a
**1436**	55.1	post	unknown	**Benign**	benign changes	n/a	n/a
**1461**	42.5	post	BRCA2+	**Benign**	benign changes	n/a	n/a
**1480**	51.3	post	BRCA2+	**Benign**	ovarian endometriosis	n/a	n/a
**1524**	55.9	post	BRCA2+	**Benign**	uterine leiomyoma	n/a	n/a
**1525**	54.3	post	LYNCH	**Benign**	cervical metaplasia	n/a	n/a
**1544**	70.7	post	BRCA2+	**Benign**	serous cystadenoma	n/a	n/a
**1556**	40.2	post	BRCA2+	**Benign**	cyst	n/a	n/a
**1573**	42.7	post	BRCA1+	**Benign**	cyst	n/a	n/a
**1575**	58.3	post	BRCA WILDTYPE	**Benign**	cyst	n/a	n/a
**1581**	71.2	post	unknown	**Benign**	serous cystadenofibroma and fibroma	n/a	n/a
**1590**	56.0	post	BRCA2+	**Benign**	benign changes	n/a	n/a
**1661**	56.4	post	BRCA1+	**Benign**	uterine adenomyosis	n/a	n/a
**1670**	56.3	post	LYNCH	**Benign**	cyst	n/a	n/a
**1731**	72.3	post	BRCA2+	**Benign**	benign changes	n/a	n/a
**1744**	51.4	post	BRCA2+	**Benign**	uterine adenomyosis, cervical metaplasia	n/a	n/a
**1758**	67.1	post	BRCA1+	**Benign**	serous cystadenoma	n/a	n/a
**1763**	57.5	post	PALB2+	**Benign**	cyst	n/a	n/a
**1790**	63.0	post	BRCA2+	**Benign**	serous adenofibroma	n/a	n/a
**1792**	52.6	post	RADBRIP1+1C+	**Benign**	cyst & uterine adenomyosis	n/a	n/a
**1817**	48.0	post	RADBRIP1+1C+	**Benign**	hydrosalpinx	n/a	n/a
**1841**	65.7	post	PALB2+	**Benign**	cyst	n/a	n/a
**1860**	53.7	post	BRCA1+	**Benign**	uterine fibroids	n/a	n/a
**1864**	67.3	post	PALB2+	**Benign**	cyst	n/a	n/a
**1870**	52.6	post	PALB2+	**Benign**	stromal hyperplasia	n/a	n/a
**1882**	51.0	post	PALB2+	**Benign**	benign changes	n/a	n/a
**1907**	67.5	post	PALB2+	**Benign**	hilar leydig cell hyperplasia	n/a	n/a
**1944**	51.5	post	RADBRIP1+1C+	**Benign**	mucinous cystadenoma	n/a	n/a
**1968**	62.9	post	RADBRIP1+1C+	**Benign**	cyst	n/a	n/a
**1987**	50.3	post	BRCA1+	**Benign**	benign changes	n/a	n/a
**2008**	55.8	post	RADBRIP1+1C+	**Benign**	uterine leiomyoma adenomyosis	n/a	n/a
**EP0003**	64.6	post	BRCA2+	**Benign**	cyst	n/a	n/a
**EP0017**	46.9	post	BRCA1+	**Benign**	fibroma and H corpus	n/a	n/a
**EP0071**	55.0	post	BRCA1+	**Benign**	uterine leiomyomata	n/a	n/a
**EP0084**	63.7	post	BRCA2+	**Benign**	cyst	n/a	n/a
**EP0099**	59.2	post	unknown	**Benign**	cyst	n/a	n/a
**EP0100**	66.5	post	BRCA2+	**Benign**	cyst	n/a	n/a
**EP0177**	55.9	post	BRCA1+	**Benign**	serous cystadenofibroma	n/a	n/a
**EP0208**	53.0	post	BRCA2+	**Benign**	fibroma	n/a	n/a
**EP0211**	58.0	post	unknown	**Benign**	serous cystadenofibromata	n/a	n/a

Sample #	AGE at collection	menopausal status	Genetic predisposition (BRCA1/2/WT/INC; LYNCH; BRIP1; RAD51C; VUS)	Benign / Malignant	Pathology type	Tumour grade	Tumour Stage
**EP0214**	50.5	post	BRCA2+	**Benign**	ovarian endometriosis and cyst	n/a	n/a
**EP0225**	58.6	post	BRCA2+	**Benign**	serous cystadenoma	n/a	n/a
**EP0305**	65.3	post	BRCA2+	**Benign**	benign changes	n/a	n/a
**EP0316**	57.1	post	BRCA2+	**Benign**	serous adenofibroma	n/a	n/a
**EP0335**	60.4	post	unknown	**Benign**	salpingitis	n/a	n/a
**EP0357**	48.3	post	unknown	**Benign**	cyst serous	n/a	n/a
**EP0372**	62.2	post	unknown	**Benign**	mucinous cystadenofibroma	n/a	n/a
**EP0384**	44.6	post	BRCA1+	**Benign**	ovarian endometriosis	n/a	n/a
**EP0390**	55.6	post	BRCA2+	**Benign**	benign changes	n/a	n/a
**EP0392**	57.0	post	BRCA2+	**Benign**	cyst	n/a	n/a
**388**	40.9	pre	BRCA WILDTYPE	**Malignant**	Granulosa cell tumour	2	1A
**464**	50.8	pre	unknown	**Malignant**	endometroid	1	1A
**845**	29.0	pre	Unknown	**Malignant**	mucinous	2	1A
**909**	50.4	pre	unknown	**Malignant**	clear cell	3	1A
**526**	60.1	post	unknown	**Malignant**	endometroid	3	1A
**894**	54.8	post	unknown	**Malignant**	serous	3	1A
**999**	60.1	post	unknown	**Malignant**	mucinous	1	1A
**1179**	53.8	post	unknown	**Malignant**	serous	3	1A
**649**	40.1	pre	unknown	**Malignant**	dysgerminoma	2	1B
**839**	40.8	pre	LYNCH	**Malignant**	endometroid / clear cell	3	1B
**56**	49.7	pre	unknown	**Malignant**	mucinous	1	1C
**136**	48.5	pre	unknown	**Malignant**	clear cell	3	1C
**262**	42.1	pre	BRCA1+	**Malignant**	clear cell	3	1C
**323**	47.8	pre	LYNCH	**Malignant**	serous / endometroid	2	1C
**844**	41.3	pre	unknown	**Malignant**	mucinous	2	1C
**350**	53.0	post	unknown	**Malignant**	endometroid	2	1C
**685**	56.7	post	unknown	**Malignant**	mucinous	2	1C
**1071**	53.9	post	BRCA1+	**Malignant**	serous	3	2A
**1197**	73.4	post	unknown	**Malignant**	serous	2	2A
**422**	68.8	post	unknown	**Malignant**	serous	3	2C
**629**	68.4	post	unknown	**Malignant**	serous	2	2C
**155**	56.0	post	BRCA2+	**Malignant**	serous	2	3A
**1077**	59.4	post	BRCA WILDTYPE	**Malignant**	serous	3	3A
**197**	49.3	pre	unknown	**Malignant**	serous	3	3B
**450**	45.1	pre	unknown	**Malignant**	serous	2	3B
**1016**	37.1	pre	BRCA1+	**Malignant**	serous	3	3B
**143**	75.3	post	Unknown	**Malignant**	serous/Primary peritoneal	3	3B
**349**	74.0	post	unknown	**Malignant**	serous	2	3B
**961**	53.0	post	BRCA WILDTYPE	**Malignant**	serous	3	3B
**10**	48.8	pre	BRCA1+	**Malignant**	serous	3	3C
**21**	52.5	pre	unknown	**Malignant**	serous	3	3C
**68**	51.5	pre	BRCA wildtype	**Malignant**	serous	3	3C
**129**	53.8	pre	unknown	**Malignant**	serous	3	3C
**147**	51.2	pre	unknown	**Malignant**	serous	3	3C
**239**	49.0	pre	Unknown	**Malignant**	serous	2	3C
**247**	46.5	pre	unknown	**Malignant**	serous	3	3C
**252**	52.4	pre	BRCA WILDTYPE	**Malignant**	serous	3	3C
**301**	49.2	pre	unknown	**Malignant**	serous / clear cell	3	3C
**317**	46.8	pre	BRCA WILDTYPE	**Malignant**	serous	3	3C
**321**	53.8	pre	BRCA WILDTYPE	**Malignant**	serous	3	3C
**336**	47.5	pre	unknown	**Malignant**	serous	2	3C
**392**	48.4	pre	BRCA2+	**Malignant**	serous	3	3C
**412**	37.4	pre	unknown	**Malignant**	adenocarcinoma poorly differentiated	3	3C
**561**	38.2	pre	BRCA1+	**Malignant**	serous	3	3C
**600**	47.6	pre	BRCA WILDTYPE	**Malignant**	serous	3	3C
**859**	39.2	pre	unknown	**Malignant**	clear cell	3	3C
**1142**	45.1	pre	VUS	**Malignant**	serous	3	3C
**7**	51.6	post	Unknown	**Malignant**	serous	3	3C
**19**	73.2	post	unknown	**Malignant**	MMMT/carcinomasarcoma	3	3C
**33**	54.0	post	BRCA WILDTYPE	**Malignant**	serous	2	3C
**37**	69.3	post	VUS (polymorphism in BRCA2)	**Malignant**	serous	3	3C
**38**	75.9	post	Unknown	**Malignant**	serous	3	3C
**40**	70.8	post	unknown	**Malignant**	serous	3	3C
**55**	76.1	post	unknown	**Malignant**	clear cell	3	3C
**58**	67.8	post	Unknown	**Malignant**	serous	3	3C
**66**	81.6	post	Unknown	**Malignant**	serous/primary peritoneal	1	3C
**70**	59.9	post	BRCA wildtype	**Malignant**	serous	2	3C
**73**	67.2	post	unknown	**Malignant**	serous	3	3C
**75**	78.2	post	unknown	**Malignant**	serous	3	3C
**83**	72.9	post	Unknown	**Malignant**	serous	3	3C
**85**	75.8	post	unknown	**Malignant**	serous	3	3C
**91**	65.2	post	Unknown	**Malignant**	serous	3	3C
**130**	68.2	post	Unknown	**Malignant**	serous	3	3C
**134**	70.1	post	Unknown	**Malignant**	serous	3	3C
**138**	71.6	post	Unknown	**Malignant**	serous	3	3C
**167**	83.2	post	Unknown	**Malignant**	serous	3	3C
**168**	65.9	post	unknown	**Malignant**	serous	3	3C
**173**	80.9	post	unknown	**Malignant**	serous	3	3C
**176**	76.8	post	unknown	**Malignant**	serous	3	3C
**181**	73.7	post	unknown	**Malignant**	serous	3	3C
**183**	60.3	post	unknown	**Malignant**	serous	3	3C
**185**	64.7	post	Unknown	**Malignant**	serous	3	3C
**186**	59.6	post	unknown	**Malignant**	clear cell	3	3C
**190**	68.6	post	unknown	**Malignant**	mucinous	2	3C
**191**	60.8	post	unknown	**Malignant**	serous	3	3C
**193**	68.7	post	unknown	**Malignant**	serous	3	3C

Sample #	AGE at collection	menopausal status	Genetic predisposition (BRCA1/2/WT/INC; LYNCH; BRIP1; RAD51C; VUS)	Benign / Malignant	Pathology type	Tumour grade	Tumour Stage
**201**	65.3	post	unknown	**Malignant**	endometroid	2	3C
**217**	73.2	post	unknown	**Malignant**	serous	3	3C
**225**	74.6	post	unknown	**Malignant**	serous	3	3C
**228**	67.5	post	unknown	**Malignant**	clear cell	3	3C
**237**	66.3	post	unknown	**Malignant**	serous	3	3C
**242**	56.5	post	Unknown	**Malignant**	serous	3	3C
**248**	75.9	post	unknown	**Malignant**	serous / endometroid	3	3C
**253**	59.1	post	unknown	**Malignant**	serous	3	3C
**254**	73.1	post	unknown	**Malignant**	serous	3	3C
**282**	55.8	post	unknown	**Malignant**	serous	3	3C
**295**	64.9	post	Unknown	**Malignant**	serous	3	3C
**307**	92.2	post	Unknown	**Malignant**	serous	3	3C
**309**	72.0	post	Unknown	**Malignant**	serous	3	3C
**339**	53.4	post	VUS	**Malignant**	serous	2	3C
**340**	62.4	post	unknown	**Malignant**	serous / clear cell	3	3C
**348**	67.8	post	unknown	**Malignant**	serous / clear cell	3	3C
**351**	65.2	post	unknown	**Malignant**	endometroid	3	3C
**361**	69.1	post	BRCA WILDTYPE	**Malignant**	serous	3	3C
**366**	63.0	post	unknown	**Malignant**	serous	3	3C
**368**	83.7	post	unknown	**Malignant**	serous / endometroid	2	3C
**389**	74.9	post	unknown	**Malignant**	serous	3	3C
**405**	84.4	post	Unknown	**Malignant**	serous	3	3C
**409**	63.7	post	Unknown	**Malignant**	serous	3	3C
**413**	63.4	post	Unknown	**Malignant**	serous	3	3C
**436**	65.1	post	unknown	**Malignant**	serous	3	3C
**452**	61.0	post	unknown	**Malignant**	serous papillary/some squamoid appearance	3	3C
**461**	67.7	post	BRCA WILDTYPE	**Malignant**	serous	3	3C
**490**	88.4	post	unknown	**Malignant**	serous	3	3C
**536**	83.9	post	unknown	**Malignant**	serous	3	3C
**552**	54.8	post	BRCA2+	**Malignant**	serous	3	3C
**590**	74.2	post	unknown	**Malignant**	serous	3	3C
**597**	67.8	post	unknown	**Malignant**	serous	3	3C
**598**	55.8	post	BRCA WILDTYPE	**Malignant**	serous	3	3C
**605**	63.1	post	unknown	**Malignant**	serous	3	3C
**606**	46.5	post	BRCA WILDTYPE	**Malignant**	serous	3	3C
**609**	63.5	post	unknown	**Malignant**	serous	3	3C
**617**	72.4	post	unknown	**Malignant**	serous	3	3C
**626**	79.9	post	unknown	**Malignant**	serous	3	3C
**630**	53.5	post	BRCA1+	**Malignant**	serous	3	3C
**650**	59.3	post	BRCA WILDTYPE	**Malignant**	serous	3	3C
**660**	67.3	post	Unknown	**Malignant**	serous	3	3C
**674**	68.8	post	BRCA WILDTYPE	**Malignant**	serous	3	3C
**677**	91.2	post	unknown	**Malignant**	serous	3	3C
**679**	69.8	post	BRCA WILDTYPE	**Malignant**	serous	1	3C
**701**	65.3	post	unknown	**Malignant**	serous	3	3C
**739**	65.4	post	BRCA2+	**Malignant**	serous	3	3C
**787**	67.1	post	unknown	**Malignant**	serous / mucinous	3	3C
**793**	62.6	post	BRCA WILDTYPE	**Malignant**	serous	3	3C
**796**	60.9	post	Unknown	**Malignant**	serous	3	3C
**803**	63.8	post	BRCA wildtype	**Malignant**	serous	3	3C
**804**	76.2	post	unknown	**Malignant**	serous	3	3C
**822**	63.8	post	unknown	**Malignant**	serous	3	3C
**829**	65.4	post	BRCA WILDTYPE	**Malignant**	serous	3	3C
**840**	67.2	post	unknown	**Malignant**	clear cell/serous	3	3C
**865**	58.2	post	unknown	**Malignant**	serous	3	3C
**870**	54.0	post	Unknown	**Malignant**	serous	3	3C
**913**	71.3	post	unknown	**Malignant**	serous	3	3C
**949**	66.2	post	unknown	**Malignant**	serous	3	3C
**950**	74.0	post	unknown	**Malignant**	serous	3	3C
**969**	61.1	post	BRCA WILDTYPE	**Malignant**	serous	3	3C
**976**	60.9	post	unknown	**Malignant**	serous	3	3C
**986**	71.0	post	unknown	**Malignant**	serous	3	3C
**1005**	85.1	post	unknown	**Malignant**	serous	3	3C
**1006**	59.5	post	unknown	**Malignant**	serous	3	3C
**1018**	76.8	post	unknown	**Malignant**	serous	3	3C
**1020**	71.0	post	BRCA WILDTYPE	**Malignant**	serous	3	3C
**1021**	70.0	post	BRCA WILDTYPE	**Malignant**	serous	3	3C
**1028**	75.2	post	unknown	**Malignant**	serous	3	3C
**1038**	69.1	post	unknown	**Malignant**	serous	3	3C
**1040**	53.7	post	unknown	**Malignant**	serous	3	3C
**1041**	68.7	post	BRCA WILDTYPE	**Malignant**	serous	3	3C
**1066**	52.1	post	BRCA2+	**Malignant**	serous	3	3C
**1067**	61.3	post	BRCA WILDTYPE	**Malignant**	serous	3	3C
**1100**	70.8	post	BRCA WILDTYPE	**Malignant**	serous	3	3C
**1101**	72.3	post	unknown	**Malignant**	serous	3	3C
**1102**	67.3	post	unknown	**Malignant**	serous	3	3C
**1107**	70.8	post	unknown	**Malignant**	clear cell	3	3C
**1110**	71.2	post	BRCA wildtype	**Malignant**	serous	3	3C
**1112**	70.6	post	unknown	**Malignant**	adenocarcinoma poorly differentiated	3	3C
**1117**	75.2	post	unknown	**Malignant**	serous	3	3C
**1124**	61.9	post	unknown	**Malignant**	adenocarcinoma poorly differentiated	3	3C
**1149**	70.7	post	BRCA2+	**Malignant**	serous	3	3C
**1154**	47.1	post	BRCA WILDTYPE	**Malignant**	serous	3	3C
**1205**	75.5	post	unknown	**Malignant**	serous	3	3C
**1221**	67.4	post	BRCA WILDTYPE	**Malignant**	serous	3	3C

Sample #	AGE at collection	menopausal status	Genetic predisposition (BRCA1/2/WT/INC; LYNCH; BRIP1; RAD51C; VUS)	Benign / Malignant	Pathology type	Tumour grade	Tumour Stage
**1234**	74.1	post	BRCA WILDTYPE	**Malignant**	serous	3	3C
**1256**	59.6	post	BRCA wildtype	**Malignant**	serous	3	3C
**394**	82.1	post	unknown	**Malignant**	Mixed MMMT/clear cell	3	4B
